# A Functional Near-Infrared Spectroscopy Examination of the Neural Correlates of Mental Rotation for Individuals With Different Depressive Tendencies

**DOI:** 10.3389/fnhum.2022.760738

**Published:** 2022-02-07

**Authors:** Liusheng Wang, Jingqi Ke, Haiyan Zhang

**Affiliations:** ^1^Teachers College, Jimei University, Xiamen, China; ^2^Institute of Special Environment Medicine, Nantong University, Nantong, China; ^3^School of Foreign Languages, Jimei University, Xiamen, China

**Keywords:** mental rotation, depression, fNIRS, psychomotor retardation, subject mental rotation, object mental rotation, mental rotation with mirrored stimuli

## Abstract

The present study aimed to examine the neural mechanisms underlying the ability to process the mental rotation with mirrored stimuli for different depressive tendencies with psychomotor retardation. Using functional near-infrared spectroscopy (fNIRS), we measured brain cortex activation of participants with higher and lower depressive tendencies while performing a left-right paradigm of object mental rotation or a same-different paradigm of subject mental rotation. Behavioral data revealed no differences in reaction time and rotation speed. The fNIRS data revealed a higher deactivation of oxyhemoglobin (HbO) change for the higher depression group in the perceptual stage of object mental rotation with mirrored stimuli in the superior external frontal cortex (BA46), inferior frontal gyrus (BA45), premotor cortex (BA6), and primary motor cortex (BA4) (study 1). In addition, there existed a significant difference between the two groups in premotor cortex (BA6) in subject mental rotation with mirrored stimuli (study 2). These results suggest that the neural mechanism of higher depression individuals connected with psychomotor retardation exists in the frontal and motor areas when processing object mental rotation with mirrored stimuli, and the motor cortex when processing subject mental rotation.

## Introduction

Individuals with depression have difficulties in emotion and cognition, presenting depressive mood for more than 2 weeks, being anhedonia, being bias toward negative information, an inhibition disorder to information, and being psychomotor retardation. According to Beck’s Unified Model of Depression (Beck and Bredemeier, 2016), depression can be viewed as an adaptation to conserve energy after the perceived loss of an investment in a vital resource such as a relationship, group identity, or personal asset. The development of processing information negatively and biological reactivity to stress mediated by alterations in brain areas/networks involved in cognition and emotion regulation leads to the negative cognitive triad; in turn, the formation and activation of the negative cognitive triad further exacerbate cognitive biases and stress reactivity. In a sense, depression is an anachronistic manifestation of an evolutionarily based “depression program” (Beck and Bredemeier, 2016), which might be connected with a specific state of the brain cortex.

Individuals with depression disorder have some specific brain cortex. The prefrontal cortex (PFC) has emerged as one of the regions most consistently impaired in major depressive disorder (Pizzagalli and Roberts, 2021). Brain structures altered in depression are part of several connectivity networks, such as in the Default Mode Network (DMN) and the Cognitive Control Network (CCN) (Gudayol-Ferré et al., 2015). Yan et al. (2019) found reduced rather than increased functional connectivity within the default mode network for major depressive disorder. In a systematic review with 68 longitudinal neuroimaging studies using fMRI and EEG, blunted reward-related (striatal) activity was a potential biological marker for both onset and course of major depression disorder (Toenders et al., 2019). Region-specific fNIR leads show patients with unipolar depression had lower hemodynamic activation in the left dorsolateral prefrontal cortex (DLPFC), orbitofrontal cortex (OFC), bilateral ventrolateral prefrontal cortex (VLPFC), and left inferior frontal gyrus (IFG) relative to healthy population (Feng et al., 2021). Some studies on depression demonstrated deactivation. Deactivation of depressive patients was more apparent in the whole brain, especially in the cingulate gyrus using fMRI (Ma et al., 2009). Patients with bipolar disorder showed more deactivation in the medial prefrontal cortex than those with unipolar depression (Rodríguez-Cano et al., 2017). Therefore, the information processing of depressive person involves the frontal cortex, and the deactivation might be presented.

Psychomotor retardation is viewed as the core of the depressive syndrome (Lemke et al., 1999), which is often assessed by means of a specific clinical rating scale (Luca et al., 2013). Mental rotation has been extensively used to gain insight into the action system of clinical populations (Oshiyama et al., 2018), such as depression, because of the advantage of mental rotation investigating the internal processes of action planning and preparation and avoiding sensory and motor confounds related to motor execution. Psychomotor retardation of depression patients was not just a symptom of emotion but a potential neurophysiological defect (Austin et al., 1999), and was associated with damage of mental rotation (Rogers et al., 2002). Psychomotor retardation was a central feature of depression that can have clinical and therapeutic implications (Bennabi et al., 2014), and may strongly influence a depressive person’s psychosocial functioning. Relative to healthy controls, depressed patients showed a generalized motor slowing (Rogers et al., 2002; Bennabi et al., 2014), and more significant temporal discrepancies on both actual and mental movements (Bennabi et al., 2014), reflecting a role of declined motor planning and prediction in the psychomotor retardation of depression patients. Mental rotation might be one better way to investigate psychomotor retardation of depressive person, being superior to some rating scales.

Mental rotation is a cognitive function whereby objects, images, or the body are mentally imaged and rotated through 2-Dimension or 3-Dimension (Shepard and Metzler, 1971). Mental rotation is a spatial representational capability of imaging or mentally rotating an object or subject. It enables one to judge contexts through viewing objects from rotated angles. Some researchers provided some arguments about the processing stages of mental rotation, including a 2-stage (Jansen et al., 2012), 3-stage (Just and Carpenter, 1976; Yan et al., 2013), and 5-stage (Corballis, 1988). Based on information processing theory and events related potentials (ERPs), Yan et al. (2013) proposed that cognitive processing of mental rotation has a perceptual stage, rotation stage and decision stage in turn. Ke and Wang (2021) found the novice drivers performed differently at perceptual and rotation stages in mental rotation about driving task. Taking superiority of processing into consideration, Jansen et al. (2012) took the reaction time in an un-rotation stage as an index of the perceptual stage, viz., the rotating angle is 0, and took the rotating speed as an index of rotation stage, viz., angle divided by corresponding reaction time. Jansen’s viewpoints have been supported by some studies (Ozel et al., 2002; Jansen et al., 2012; Feng et al., 2019; Ke and Wang, 2021), supporting that the rotating speed is a better index over the correct rate. Neuroimaging studies on healthy populations revealed activation of specific brain regions recruited spatial cognitive processing, such as frontal (BA for Brodmann Area, BA9, BA10), premotor (BA6), parietal cortex (BA40, BA44) (Hyun and Luck, 2007). Further, the areas of activation in mental rotation were premotor area, bilateral superior parietal lobule and visual extrastriate cortex (Vingerhoets et al., 2002). Some neuroscience studies have identified brain regions activated in the frontal cortex (BA9, BA44), premotor cortex (BA6) and parietal cortex (BA40), basal ganglia, cerebellum during mental rotation (Jordan et al., 2001; Schöning et al., 2007). Using fNIRS, increased oxyhemoglobin (HbO) in BA44 was observed in high and low-performance groups. In contrast, a decrease in deoxyhemoglobin (HbR) in BA9 was observed in only the high-performance group and BA44 only in the low-performance group. The BA44 is considered one of the core neural correlates of mental rotation, while BA6 and BA9 might be compensatory (Wu et al., 2020). Thus, the frontal cortex and motor cortex might participate in the mental rotation processing, especially involving the BA6, BA9, BA40, and BA44.

Mental rotation has object and subject mental rotation according to the stimuli type of task, with mirrored or identical (normal) stimuli. An object, such as letter, digit, 3-D picture and true scene, or subject, such as body of person and body parts (hand, arm, leg, and face), is mentally rotated in mental rotation, referring to object mental rotation or subject mental rotation, respectively (Dalecki et al., 2012; Chen and Hu, 2019). Chen et al. (2013a) found mental rotation deficits specific to the hand than the letter task, suggesting that the mental imagery for hands and letters relies on different processing mechanisms. Compared to the healthy controls, depression patients demonstrated a lower error rate, shorter reaction time and larger P500 aptitude in mirror image mental rotation, reflecting an impairment of information processing of representation rotating in patients with depressive disorder (Chen et al., 2013b,c). Furthermore, some ERPs studies showed that mirror-normal difference in both the early and late phases of mental rotation and deduced that flipping is more likely to occur in the late phase of the mirror-normal letter discrimination task (Quan et al., 2017), and hypoxia effect was effective with normal letters but had little effect on the mirrored letters (Ma et al., 2016). A Beta-band ERD Study found the temporal difference of beta ERD between the identical and the mirrored stimuli at 0°rotation and the ERD topographic difference in left fronto-parietal regions (Chen et al., 2014). A self-paced event-related fMRI design revealed the differences in visual cortex activation between tasks with mirrored and identical figures (Paschke et al., 2012). So, processing deficits related to object mental rotation with depression might be different from that of subject mental rotation, and it is necessary to differentiate mirrored from identical tasks in mental rotation experiment.

Usually, different experiment paradigms are employed to different kinds of mental rotation. A left-right paradigm (L-R paradigm) is often conducted in object mental rotation in which the original and mirrored images are simultaneously set on the left and right parts of the screen, respectively, and participants are required to judge the similarity of two images; a same-different paradigm (S-D paradigm) is used in subject mental rotation in which two kinds of stimuli present separately in the order, and participants are asked to judge whether the target stimulus is the same as the original stimulus stayed in the short memory (Jolicocur et al., 1987; Kosslyn et al., 2001; Dalecki et al., 2012; Chen and Hu, 2019). The nature of image stimulus in the mental rotation might be the key to lead to the activation of motor area; when the stimulus was subject, the motor cortex (M1) was activated, and premotor cortex was also activated for half of participants (Kosslyn et al., 1998, 2001).

Relative to the other techniques including EEG, fMRI, and PET, fNIRS has some advantages. fNIRS is a non-invasive optical imaging technique that measures changes in hemoglobin (Hb) concentrations within the brain by means of the characteristic absorption spectra of Hb in the near-infrared range. fNIRS systems are relatively insensitive to participant motion and have a portable, compact, and increasingly miniaturized design (Di Domenico et al., 2019). Many studies support the reliability of fNIRS to measure the cerebral hemodynamics in spatial cognitive tasks (Herff et al., 2013; Fu et al., 2016). Unlike EEG or ERPs being heavily dependent on the superposed average of data, fNIRS focuses the data during a period of time. Therefore, relatively small sample size (Li et al., 2019; Mauri et al., 2020; Zhang et al., 2020), and relatively small trial size could be acceptable (Du et al., 2015). The fNIRS itself provides a better way to ascertain the processing of psychomotor retardation of depression in mental rotation, being better than scales or surveys.

The current study aimed to examine the relevant activation regions recruited mental rotation with mirrored stimuli for depressive tendencies individuals with psychomotor retardation. Here are two research questions. The research question 1 is that, is there differences of activation regions, mainly frontal and motor cortex, between different depressive tendencies in mental rotation. The research question 2 is that, is there any differences of activation regions above mentioned between object and subject mental rotation. Two experiments were conducted through fNIRS technique and a left-right paradigm (study 1) or a same-different paradigm (study 2) of mental rotation, in order to answer these two research questions. One factor between-subjects design was employed (Yan et al., 2021), with depressive tendency as independent variable. Two experiments were object mental rotation (Study 1) and subject mental rotation (Study 2), using mirrored stimuli, taking reaction time (Rt) at angle 0° as behavior index of the perceptual stage (stimulus encoding) and rotating speed as an index of the rotating stage (responsive to motor speed), taking beta of HbO change in brain areas as an index of biology. Taking the angle of rotation and reaction time into consideration, rotating speed is a sensitive index in measuring and assessing mental rotation (Ozel et al., 2002; Jansen et al., 2012; Feng et al., 2019; Ke and Wang, 2021). Based on the literature of mental rotation and depression patients, the overlapping cortex lies in frontal and motor cortex, the hypothesis 1 corresponding to the research question 1 is that, the differences of activation regions between different depressive tendencies in mental rotation could exists in frontal and motor cortex. According to the literature of object and subject mental rotation, the hypothesis 2 corresponding to the research question 2 is that, there would exist difference of activation regions above mentioned between object and subject mental rotation for depressive individuals.

## Study 1

This experiment investigated the difference in activation areas recruited mirror movement in object mirror mental rotation between different depressive tendencies.

### Method

#### Participants

A total of 41 college students participated in the experiment. All were right-handed, reported no brain or psychology illness history, normal version or corrected-version. Forty participants were analyzed, aged mean 23.63, *SD* = 1.90, one participant was excluded whose fNIRS signal record failed.

#### Materials and Tools

**FIGURE 1:**
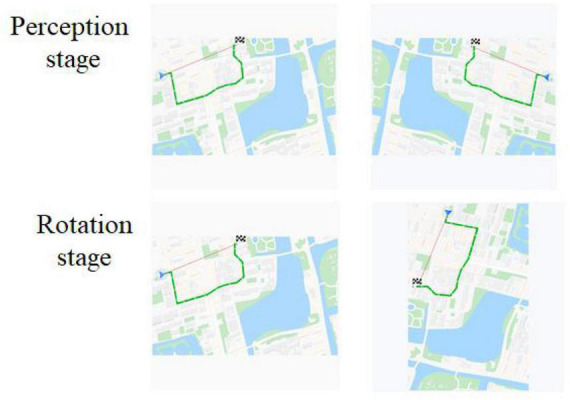
Experimental Materials for object mental rotation. The first column was original images, and the second column was rotation or mirror images.

Object mirror images were GPS map by Photoshop2018 software, referred to map elements (Mei, 2011), and Baidu map (see Figure 1). These mirror images were generated at a spatial rotation angle of 0°, 90°, 180°, 270°, of them, 0° belonging to the perceptual stage, 90°, 180°, and 270° belonging to rotation stage.

BDI-II-C (Beck Depression Inventory, BDI) was a Chinese version of Beck Depression Inventory (Beck et al., 1996; Wang et al., 2011; Yang et al., 2014), applied to self-assess depression symptoms in the past 2 weeks, including 21 items.

#### Procedure

**FIGURE 2:**
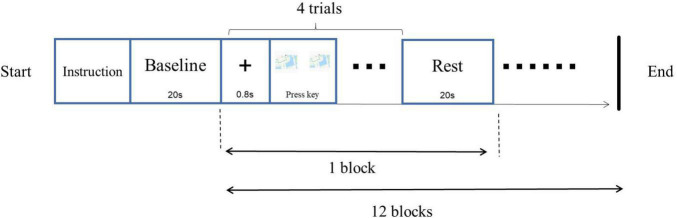
Experimental procedure of object mental rotation.

A left-right paradigm (L-R paradigm) was employed for this object mental rotation. The procedure was programmed with E-prime 3.0 software and presented on a 19-inch computer. Screen resolution was 1,440 × 900, and the refresh rate was 75 Hz. Before the experiment, the participant wore a fiber cap, and the equipment was calibrated. In addition, a 3-D head model was constructed. The procedure was as followed (Figure 2). After the instruction, the fixation “+” was on the middle of the screen for 800 ms, followed by two images on the left and right parts of the screen. The participant was required to judge whether these two images were the same if the images were rotated, pressed key “F” for the same, pressed key “J” for the difference. The style of key-pressed was counterbalanced. There were 48 trials (12 blocks), of which 24 trials were mirror images. The rest time for every block was 20 s.

#### Collecting and Analysis of fNIRS Data

**FIGURE 3:**
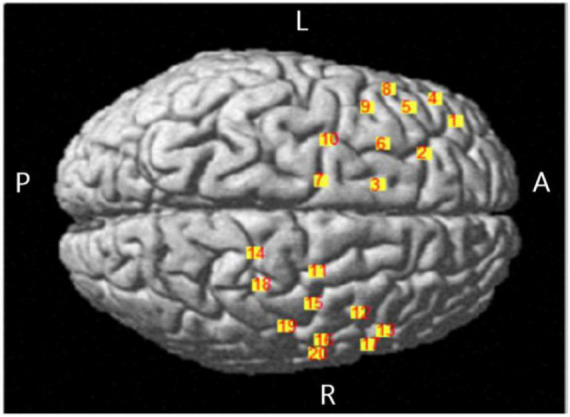
Location map of brain projection of each channel.

The equipment of fNIRS with LABNIRS system was from Japan Shimadzu corporation, recording the concentration changes of oxygenated hemoglobin (HbO). There were 16 optrodes set in 10–20 systems, and there were 20 channels (Figure 3 and Table 1). 3D-digitizer positioned the coordinates of all channels and the areas of the brain include: (a) dorsolateral prefrontal cortex (DLPFC and BA9) corresponding to channels 2, 5, and 9; (b) superior external frontal cortex (BA46) corresponding to channels 1; (c) premotor cortex (BA6 and BA8) corresponding to channels 3, 6, 7, 10, 11, 12, 15, 16, and 20; (d) inferior frontal gyrus (IFG, BA44, and BA45) corresponding to channels 4, 8, 13, and 17; and (e) the primary motor cortex (BA4) corresponding to channels 14, 18, and 19.

**TABLE 1:** Position of all fNIRS channels.

Channel	Montreal neurological institute			Brodmann area	
	x	y	z	Anatomical label	Percentage of overlap
CH1	−39	47	31	46-superior external frontal cortex	0.73
CH2	−27	37	51	9-dorsolateral prefrontal cortex	0.74
CH3	−12	17	70	6-premotor cortex	0.70
CH4	−50	39	23	45-inferior frontal gyrus	1.00
CH5	−46	30	42	9-dorsolateral prefrontal cortex	0.36
CH6	−29	19	62	8-premotor cortex	0.86
CH7	−14	−5	76	6-premotor cortex	1.00
CH8	−54	22	33	44-inferior frontal gyrus	0.74
CH9	−46	14	54	9-dorsolateral prefrontal cortex	0.61
CH10	−31	−2	68	6-premotor cortex	1.00
CH11	26	−6	73	6-premotor cortex	1.00
CH12	44	9	59	6-premotor cortex	0.58
CH13	52	20	42	44-inferior frontal gyrus	0.61
CH14	19	−29	78	4-primary motor cortex	0.85
CH15	40	−9	68	6-premotor cortex	0.79
CH16	56	−4	53	6-premotor cortex	0.77
CH17	59	15	34	44-inferior frontal gyrus	0.70
CH18	33	−29	74	4-primary motor cortex	0.72
CH19	51	−18	63	4-primary motor cortex	0.57
CH20	61	−7	46	6-premotor cortex	0.59

Data of fNIRS were analyzed using NIRS_SPM software in Matlab (R2014b), which usually run based on a general linear model (GLM) (Ye et al., 2009). The current study mainly analyzed HbO, the most sensitive parameter to local brain blood and correlated to blood signals. The procedure of data analysis was as follows. Step 1 dealt with the noise elimination of raw data using the hemodynamic response function (HRF) and wavelet-minimum description length (wavelet-MDL). Step 2, parameter estimation, was taken through a GLM model to get beta value under every condition. Then, the beta was baseline-corrected in which ΔHbO2 of each channel was calculated as the difference between the beta of task-stage and the beta of baseline after 15 s. The positive beta meant activation, while minus beta meant deactivation (Rodrigo et al., 2014). Both were the state of brain activation. Step 3, the beta was analyzed and corrected using false discovery rate (FDR). FDR is usually used to correct the *p*-values of fNIRS data (Benjamini and Hochberg, 1995; Singh and Dan, 2006; Bai et al., 2016; Zhang et al., 2019).

### Results

The raw score of BDI-II-C was transferred into a Z score. Twenty-seven participants were assigned to the lower depression tendencies group whose Z score was less than 0, while 13 participants were in the higher group whose Z score was greater than 0. The independent samples *t*-test showed that the raw score of BDI in the higher group (*M* = 11.38, *SD* = 7.01) was significantly greater than that in the lower group (*M* = 1.74, *SD* = 1.56), *t*(38) = 6.895, *p* < 0.001. Taking reaction time at angle 0° (non-rotation) as behavior index of the perceptual stage (stimulus encoding), and rotating speed as an index of the rotating stage which was mean of a ratio of angle and corresponding reaction time (Jansen et al., 2012), viz., the formula was, rotation speed $=\left(\frac{90}{R\,T\,90}+\frac{180}{R\,T\,180}+\frac{270}{R\,T\,270}\right)\,/\,3$.

The independent samples *t*-test showed no significant difference in the reaction time of the perceptual stage and rotation speed of the rotation stage (upper part of Table 2), ts < 1.

**TABLE 2:** The comparison between two groups in reaction time and rotation speed.

Variables	Depression	*M*	*SD*	*t*
Rt of perceptual stage for object mirror images (ms)	Lower	4148.82	2357.90	–0.512
	Higher	4576.64		
Rotation speed of rotation stage for object mirror images (°/s)	Lower	44.83	24.76	0.711
	Higher	39.29		
Rt of perceptual stage for subject mirror images (ms)	Lower	1275.13	550.10	–0.183
	Higher	1312.24		
Rotation speed of rotation stage for subject mirror images (°/s)	Lower	49.57	17.73	–0.496
	Higher	52.79		

The independent samples *t*-test was conducted to examine the differences of beta values of HbO change in different channels in perceptual stage (Table 3), rotation stage (Table 4), between lower and higher groups. The results showed, HbO change in the higher depression group was smaller than that in the lower group in five channels, including channel 1 [*t*(38) = 3.013, *d* = 1.02, *p* < 0.01], channel 4 [*t*(38) = 2.813, *d* = 0.95, *p* < 0.01], channel 7 [*t*(38) = 3.174, *d* = 1.07, *p* < 0.01], channel 11 [*t*(38) = 2.157, *d* = 0.73, *p* < 0.05], and channel 14 [*t*(38) = 2.034, *d* = 0.69, *p* < 0.05], in perceptual stage.

**TABLE 3:** The comparison of brain activation between higher and lower groups in perceptual stage for object mirrored images (HbO).

Channels	BA	Depression	*M*	*SD*	*t*	*p*
CH1	46	Lower	–0.000028	0.004480	3.013**	0.005
		Higher	–0.007783	0.011859		
CH2	9	Lower	–0.000364	0.009535	–0.569	0.573
		Higher	0.002156	0.018668		
CH3	6	Lower	0.000960	0.005003	1.648	0.108
		Higher	–0.001993	0.005908		
CH4	45	Lower	0.000977	0.004204	2.813**	0.008
		Higher	–0.003272	0.005008		
CH5	9	Lower	0.002658	0.013941	0.753	0.456
		Higher	–0.000459	0.007441		
CH6	8	Lower	0.001936	0.014181	–0.233	0.817
		Higher	0.003319	0.023310		
CH7	6	Lower	0.000099	0.004509	3.174**	0.003
		Higher	–0.006067	0.007799		
CH8	44	Lower	0.000648	0.005234	0.438	0.664
		Higher	–0.000063	0.003740		
CH9	9	Lower	–0.001198	0.016707	0.974	0.336
		Higher	–0.006065	0.009440		
CH10	6	Lower	–0.000523	0.015781	–0.269	0.789
		Higher	0.000815	0.012179		
CH11	6	Lower	0.001565	0.004576	2.157*	0.037
		Higher	–0.001664	0.004111		
CH12	6	Lower	0.000033	0.005373	1.196	0.236
		Higher	–0.001910	0.003287		
CH13	44	Lower	0.001311	0.005431	1.299	0.202
		Higher	–0.000791	0.002973		
CH14	4	Lower	0.000152	0.004210	2.034*	0.049
		Higher	–0.002739	0.004213		
CH15	6	Lower	0.004230	0.022366	0.611	0.545
		Higher	0.000364	0.005416		
CH16	6	Lower	–0.000708	0.008389	0.655	0.517
		Higher	–0.002327	0.004194		
CH17	44	Lower	0.003042	0.007425	0.622	0.538
		Higher	0.001611	0.005258		
CH18	4	Lower	0.000780	0.004720	–0.144	0.886
		Higher	0.001166	0.012272		
CH19	4	Lower	0.003370	0.014454	0.756	0.454
		Higher	0.000222	0.005428		
CH20	6	Lower	0.001693	0.012112	0.975	0.336
		Higher	–0.001662	0.003331		

*p < 0.05, **p < 0.01. The same below.

**TABLE 4:** The comparison of brain activation between higher and lower groups in rotation stage for object mirrored images (HbO).

Channels	BA	Depression	*M*	*SD*	*t*	*p*
CH1	46	Lower	0.004035	0.015453	1.690	0.099
		Higher	–0.003679	0.007823		
CH2	9	Lower	0.001117	0.013338	1.207	0.235
		Higher	–0.003791	0.008595		
CH3	6	Lower	0.000930	0.011024	1.525	0.136
		Higher	–0.003956	0.004676		
CH4	45	Lower	0.004519	0.017675	1.407	0.168
		Higher	–0.002765	0.008265		
CH5	9	Lower	0.003816	0.013862	1.537	0.133
		Higher	–0.002264	0.004328		
CH6	8	Lower	0.005185	0.015301	1.967	0.056
		Higher	–0.003685	0.007587		
CH7	6	Lower	–0.000682	0.009057	1.499	0.142
		Higher	–0.005340	0.009514		
CH8	44	Lower	0.003123	0.018742	0.473	0.639
		Higher	0.000626	0.003474		
CH9	9	Lower	–0.001053	0.015228	0.672	0.506
		Higher	–0.004718	0.018029		
CH10	6	Lower	0.000733	0.016803	0.210	0.835
		Higher	–0.000336	0.010297		
CH11	6	Lower	0.003844	0.011945	1.182	0.245
		Higher	–0.000161	0.003165		
CH12	6	Lower	0.004039	0.012829	1.394	0.171
		Higher	–0.001116	0.004810		
CH13	44	Lower	0.006036	0.018372	1.213	0.233
		Higher	–0.000227	0.003178		
CH14	4	Lower	0.003669	0.012527	1.676	0.102
		Higher	–0.002378	0.004682		
CH15	6	Lower	0.004112	0.012710	1.164	0.252
		Higher	–0.000095	0.003636		
CH16	6	Lower	0.005703	0.017282	1.322	0.194
		Higher	–0.000812	0.005314		
CH17	44	Lower	0.006692	0.019416	0.446	0.658
		Higher	0.004213	0.006529		
CH18	4	Lower	0.003864	0.013504	1.556	0.128
		Higher	–0.002111	0.003802		
CH19	4	Lower	0.003850	0.013224	1.345	0.186
		Higher	–0.001225	0.004062		
CH20	6	Lower	0.005059	0.019985	0.870	0.390
		Higher	0.000164	0.003887		

**FIGURE 4:**
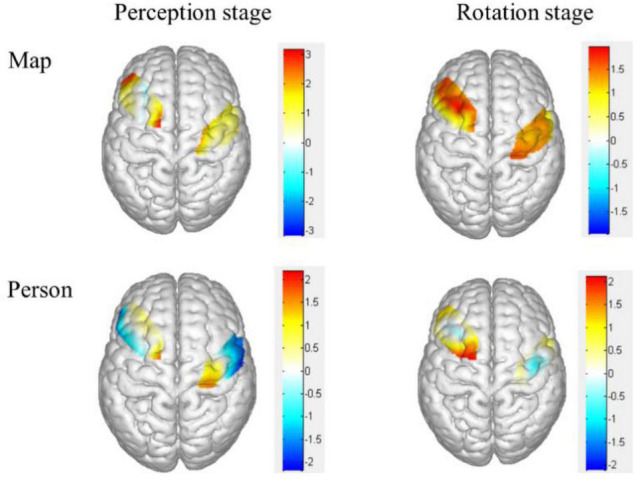
Heat map of *t*-values for the comparison of different groups of brain activation in different stages. The upper was the object mirror images, and the bottom was the subject mirror images. The bigger the two groups, the thicker the color.

The brain activation map of higher and lower groups was drawn based on *t*-values of channels (upper part of Figure 4). Specifically, the heat map of *t*-values of object mirror images in the perception stage covered channels 1, 4, 7, 11, and 14, corresponding to the superior external frontal cortex (BA46), inferior frontal gyrus (BA45), premotor cortex (BA6), the primary motor cortex (BA4), indicating a more substantial deactivation of the higher group.

### Discussion

The experiment of study 1 found the deactivation areas recruited in object mental rotation with mirrored stimuli for higher depressive tendencies group are located in the superior external frontal cortex (BA46), inferior frontal gyrus (BA45), premotor cortex (BA6), the primary motor cortex (BA4), relative to lower group, indicating the frontal and motor areas related with psychomotor retardation of depression. These two areas execute the mental rotation with psychomotor retardation, co-functioning the inhibition and motor.

## Study 2

This experiment investigated the difference in activation areas recruited mirror movement in subject mental rotation between different depressive tendencies.

### Method

**FIGURE 5:**
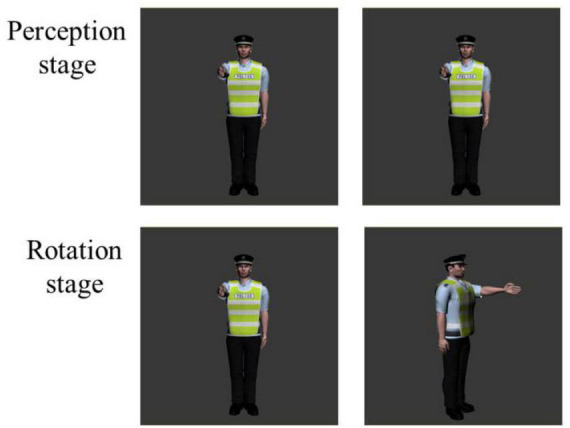
Experimental Materials for subject mental rotation. The first column were original images, and the second column were rotation or mirrored images.

**FIGURE 6:**
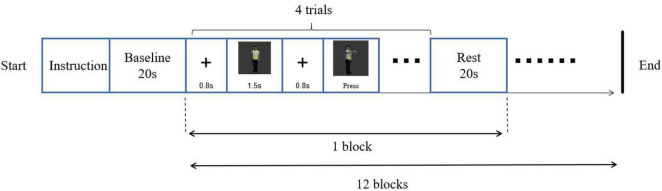
Experimental procedure of subject mental rotation.

Participants, tools, procedure, method of fNIRS data collecting and analysis in study 2 were the same as those in study 1. Materials were subject images of traffic police taking eight gestures (Figure 5). Person images were rotated at angles of 0°, 30°, 60°, and 90°. A same-different paradigm (S-D paradigm) was employed for this subject mental rotation. The method of stimuli presented in S-D paradigm was different from the L-R paradigm of study 1, in which the images of original image was presented before, then the rotation or mirrored image came out in S-D paradigm (Figure 6).

### Results

Regarding the behavior data, the independent samples *t*-test showed no significant difference in reaction time of the perceptual stage and rotation speed of the rotation stage (see Table 2), ts < 1.

Concerning the fNIRS data, the independent samples *t*-test was conducted to examine the differences of beta values in different channels in perceptual stage (Table 5), rotation stage (Table 6), between lower and higher groups. The results showed, changes of oxygenated hemoglobin (HbO) in higher depression group was bigger than that in lower group in channel 20 in perceptual stage [*t*(38) = -2.178, *d* = 0.74, *p* < 0.05], and smaller in the rotation stage in channel 7 [*t*(38) = 2.106, *d* = 0.71, *p* < 0.05] and channel 10 [*t*(38) = 2.089, *d* = 0.55, *p* < 0.05].

**TABLE 5:** The comparison of brain activation between higher and lower groups in perceptual stage for subject mirrored images (HbO).

Channels	BA	Depression	*M*	*SD*	*t*	*p*
CH1	46	Lower	0.001613	0.005026	0.429	0.670
		Higher	0.000941	0.003662		
CH2	9	Lower	–0.000353	0.012355	0.625	0.539
		Higher	–0.003017	0.013221		
CH3	6	Lower	0.000089	0.004126	0.277	0.783
		Higher	–0.000282	0.003611		
CH4	45	Lower	0.001005	0.004748	-1.950	0.059
		Higher	0.004983	0.008172		
CH5	9	Lower	0.000435	0.004009	-0.084	0.933
		Higher	0.000560	0.005174		
CH6	8	Lower	–0.000389	0.010852	0.259	0.797
		Higher	–0.001651	0.020118		
CH7	6	Lower	0.001446	0.005873	2.017	0.051
		Higher	–0.002649	0.006308		
CH8	44	Lower	0.000883	0.005603	-1.620	0.114
		Higher	0.003661	0.003700		
CH9	9	Lower	–0.001700	0.010358	-1.301	0.201
		Higher	0.002456	0.007156		
CH10	6	Lower	0.001411	0.013798	-0.553	0.584
		Higher	0.003797	0.010286		
CH11	6	Lower	0.001300	0.003691	0.701	0.487
		Higher	0.000447	0.003403		
CH12	6	Lower	–0.001504	0.008025	-1.047	0.302
		Higher	0.001031	0.004831		
CH13	44	Lower	–0.001289	0.006352	-1.626	0.112
		Higher	0.001882	0.004283		
CH14	4	Lower	0.001304	0.003498	1.420	0.164
		Higher	–0.000388	0.003595		
CH15	6	Lower	0.000783	0.005555	0.505	0.616
		Higher	–0.000147	0.005217		
CH16	6	Lower	0.000166	0.005418	-1.491	0.144
		Higher	0.002809	0.004872		
CH17	44	Lower	0.000758	0.005475	-1.662	0.105
		Higher	0.004215	0.007440		
CH18	4	Lower	0.001252	0.003565	1.665	0.104
		Higher	–0.003990	0.015742		
CH19	4	Lower	0.000154	0.005429	-1.756	0.087
		Higher	0.003505	0.006106		
CH20	6	Lower	–0.001511	0.006689	-2.178*	0.036
		Higher	0.003294	0.006186		

*p < 0.05.

**TABLE 6:** The comparison of brain activation between higher and lower groups in rotation stage for subject mirrored images (HbO).

Channels	BA	Depression	*M*	*SD*	*t*	*p*
CH1	46	Lower	0.004325	0.012576	1.280	0.208
		Higher	–0.001288	0.013844		
CH2	9	Lower	0.002555	0.023724	0.301	0.765
		Higher	0.000536	0.005621		
CH3	6	Lower	0.003322	0.008948	0.919	0.364
		Higher	0.000871	0.004895		
CH4	45	Lower	0.005618	0.013055	1.402	0.169
		Higher	–0.000570	0.013109		
CH5	9	Lower	0.003846	0.009551	-0.187	0.853
		Higher	0.004424	0.008195		
CH6	8	Lower	0.001118	0.026158	-0.385	0.702
		Higher	0.004382	0.022609		
CH7	6	Lower	0.004895	0.008525	2.106*	0.042
		Higher	–0.001432	0.009665		
CH8	44	Lower	0.007027	0.016478	0.606	0.548
		Higher	0.004058	0.008808		
CH9	9	Lower	0.004932	0.019599	0.496	0.623
		Higher	0.002190	0.004053		
CH10	6	Lower	0.011728	0.019720	2.089*	0.044
		Higher	0.002478	0.008224		
CH11	6	Lower	0.004446	0.007031	0.556	0.581
		Higher	0.003273	0.004089		
CH12	6	Lower	0.002763	0.013599	0.107	0.915
		Higher	0.002330	0.007357		
CH13	44	Lower	0.004629	0.012235	0.879	0.385
		Higher	0.001575	0.003373		
CH14	4	Lower	0.004917	0.008615	-0.011	0.991
		Higher	0.004950	0.009409		
CH15	6	Lower	0.003937	0.006436	-0.878	0.386
		Higher	0.006248	0.010139		
CH16	6	Lower	0.001224	0.023450	-0.189	0.851
		Higher	0.002473	0.005447		
CH17	44	Lower	0.006499	0.012578	0.752	0.457
		Higher	0.003701	0.006456		
CH18	4	Lower	0.004397	0.007741	0.147	0.884
		Higher	0.004040	0.005788		
CH19	4	Lower	0.002713	0.010935	0.053	0.958
		Higher	0.002545	0.004808		
CH20	6	Lower	0.003256	0.009446	0.232	0.817
		Higher	0.002607	0.004818		

*p < 0.05.

The brain activation map of higher and lower groups was drawn based on *t*-values of channels (bottom part of Figure 4). The *t*-values hot contrast images of subject mirror images in the perception stage covered channel 20 corresponding to the premotor cortex (BA6), in rotation stage channel 7 and 10 corresponding to the premotor cortex (BA6).

### Discussion

The experiment of study 2 found the deactivation area recruited in subject mental rotation with mirrored stimuli for higher depressive tendencies group lies in the premotor cortex (BA6) in perceptual stage, and the activation in the premotor cortex (BA6) in rotation stage, relative to lower group, indicating only motor areas related with psychomotor retardation of depression. It is different from the results of object mental rotation in study 1 in which the frontal and motor cortex were recruited for higher group. Subject mental rotation only involves the premotor cortex (BA6).

## General Discussion

This research mainly found a higher deactivation of changes of oxygenated hemoglobin (HbO) for higher depressive tendency participants in object mental rotation with mirrored stimuli in the superior external frontal cortex (BA46), inferior frontal gyrus (BA45), premotor cortex (BA6), the primary motor cortex (BA4), indicating a unique role of the frontal cortex and motor cortex in mental rotation processing of depression individual. These findings could correspond the research question 1 and evidences the hypothesis 1. Mental rotation is a spatial representation located in the functional areas, including the premotor cortex and primary motor cortex (Jordan et al., 2001; Vingerhoets et al., 2002; Hyun and Luck, 2007; Hétu et al., 2013). The deactivation of the frontal cortex is recruited in spatial cognitive processing for depression patients (Hyun and Luck, 2007; Rodríguez-Cano et al., 2017; Feng et al., 2021), which is consistent with the founding of the current study. Psychomotor retardation of depression associated with damage of mental rotation (Austin et al., 1999; Rogers et al., 2002), did not come out by way of an explicit index, such as reaction time and rotation speed in this study, supporting “depression program” in Beck’s Unified Model of Depression (Beck and Bredemeier, 2016).

Deactivation is the metabolism attenuation of cerebral blood flow during cognitive task processing. It is unconscious and cannot be aware. That is to say, significant increases in oxyhemoglobin (HbO) compared to baseline were observed in the task of processing mental rotation, and brain blood flow, level of HbO and local brain nerve activity would attenuate. The function of the deactivation area is to focus on the attention and to allocate the attention resource automatically. Deactivation of the corresponding brain area would come out, when individuals pay attention to execute one task which reduces the activity of extensive information collection, and the level of activation lowers down (Feng et al., 2007; Li and Shu, 2014). Deactivation might function as inhibition or monitoring of some information in which some redundancy information can be filtered to amplify useful information (Drevets et al., 1995). The frontal cortex is a default mode network connected with control of cognition and monitoring tasks, especially in representing target maintenance and inhibition (Paxton et al., 2008; Yan et al., 2019). Prefrontal cortex deactivation means a control strategy to cognitive resources to represent cue information (Zhang et al., 2020). Some fNIRS studies reported deactivation of dorsolateral prefrontal cortex (DLPFC) and ventrolateral prefrontal cortex (VLPFC) during inhibition control (Nishimura et al., 2011; Wriessnegger et al., 2012; Rodrigo et al., 2014). When depressive individuals process mental rotation, cognitive resources need to reassign, leading to a bigger HbO change, consistent with the findings by Shmuel et al. (2002).

Another finding is the difference between object and subject mental rotation with mirrored stimuli for higher depressive tendency individuals, corresponding to the research question 2 and supporting the hypothesis 2, indicating a different processing mechanism between object and subject mental rotation for depressive individuals. Compared to the lower depressive group, the higher group recruits the frontal cortex to process the object mental rotation relative to subject mental rotation, including superior external frontal cortex (BA46) and inferior frontal gyrus (BA45). This result is in line with one mental rotation study of healthy population (Vingerhoets et al., 2002). One explanation is about the stimuli types in different paradigms of object and subject mental rotation which affect the spatial representation (Kosslyn et al., 1998, 2001). The task of L-R paradigm in object mental rotation (i.e., study 1) includes the searching, transforming, comparing and confirming (Shepard and Metzler, 1971), the task of S-D paradigm in subject mental rotation (i.e., study 2) mainly focuses judging rotation (Jolicocur et al., 1987). In a sense, the former requires more monitoring and translation which are function of frontal cortex, comparing with the latter. Alternative explanation is about embodied cognition. The activation of cortical regions during mental rotation seems at least in part determined by an intrinsic process that depends on the afforded actions elicited by the kind of stimuli presented. More studies of healthy population using ERP, ERD, or fMRI techniques found a different neural mechanism between object and subject mental rotation (Paschke et al., 2012; Chen et al., 2014; Ma et al., 2016; Quan et al., 2017). Further, Chen et al. (2013b,c) reported that patients with depressive disorder had an impairment processing mental rotation with mirrored stimuli. In the perceptual stage of mental rotation with mirrored stimuli, participants need to imagine themselves in different angles, embodied themselves in the object (map) or subject (person) position. A study on a healthy population demonstrates a more vital link between the bodily self and motor representations (Kaltner et al., 2014). The extent of embodiment in an object is more complex than that in a subject (the body of a person) (Barsalou, 2008; Wang and Sang, 2014). Using human bodies as stimulus material elicits embodied spatial transformations (Kaltner et al., 2014). The human body (i.e., traffic policeman in study 2), not object (i.e., map in study 1), induces embodied spatial representation. Thus, the specific brain area of monitoring and inhibition, i.e., frontal brain areas, would be recruited for participants of the higher group to execute the object mental rotation.

Other, there were no significant differences between higher and lower groups in the reaction time of the perceptual stage and rotation speed of the rotation stage in both object and subject mental rotation. It seems that the results of these behavioral indices are not consistent with the results of fNIRS data. Here is one explanation from the perspective of nature of two kinds of measure data. Behavioral data focus the behavioral results of whole processing which includes the different stages of processing. The fNIRS data focus the brain tissue concentration changes in oxyhemoglobin (HbO2) and deoxyhemoglobin (HbR) associated with an increased metabolic demand of the brain during neuronal activity (Pinti et al., 2019), presented during a certain time period. The latter is more accurate and is out of the control of consciousness.

In sum, our work was the first study to uncover the underlying neural mechanisms of psychomotor retardation of depressive individuals measured by mental rotation using the fNIRS method. It is concluded that the neural areas of higher depressive tendency individuals connected with psychomotor retardation exists in the frontal and motor cortex when processing object mental rotation, and the motor cortex when processing subject mental rotation.

## Data Availability Statement

The original contributions presented in the study are included in the article/Supplementary Material, further inquiries can be directed to the corresponding author.

## Ethics Statement

The studies involving human participants were reviewed and approved by the Nantong University Committee. The patients/participants provided their written informed consent to participate in this study.

## Author Contributions

LW and HZ developed the study concept, contributed to the design, and wrote and revised the manuscript. LW and JK implemented the experiment, and collected and analyzed the data. All authors contributed to the article and approved the submitted version.

## Conflict of Interest

The authors declare that the research was conducted in the absence of any commercial or financial relationships that could be construed as a potential conflict of interest.

## Publisher’s Note

All claims expressed in this article are solely those of the authors and do not necessarily represent those of their affiliated organizations, or those of the publisher, the editors and the reviewers. Any product that may be evaluated in this article, or claim that may be made by its manufacturer, is not guaranteed or endorsed by the publisher.
